# Supplementary material for: The evolution of olfactory sensitivity, preferences, and behavioral responses in Mexican cavefish is influenced by fish personality
Source: eLife. 2024 Jun 4;12:RP92861. doi: 10.7554/eLife.92861 (PMC11149931; doi:10.7554/eLife.92861)
Supplement: Supplementary file 1. — (A) Population level summary. For each amino acid and each concentration tested, arrows indicate whether the considered parameter has changed (increased or decreased). ns indicates no significant change, - indicates the condition was not tested in the fish type (SF in blue, CF in red, F2 in green). (B) Individual level summary. The percentage of fish displaying an individual olfactory score superior to 1.5 is indicated. [file elife-92861-supp1.pdf]

Supplementary File 1

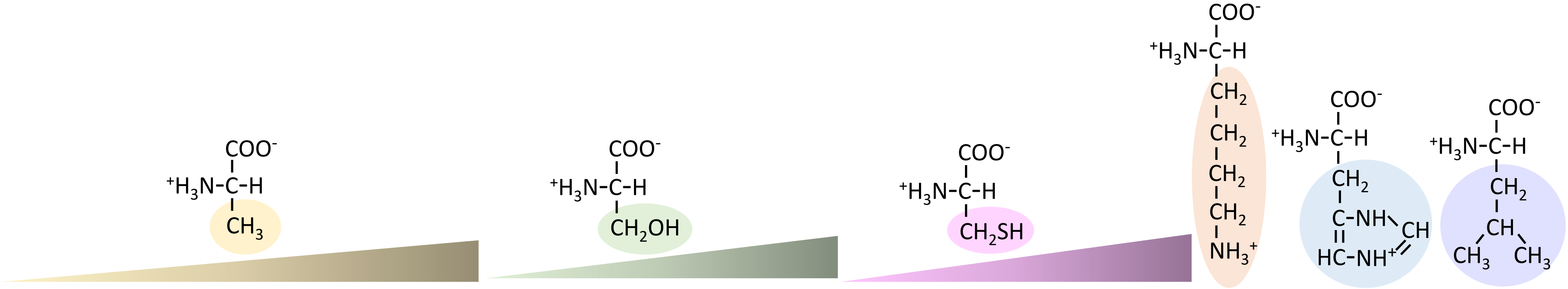

	alanine 10^{-5}M	alanine 10^{-4}M	alanine 10^{-3}M	alanine 10^{-2}M	serine 10^{-4}M	serine 10^{-3}M	serine 10^{-2}M	cysteine 10^{-4}M	cysteine 10^{-3}M	cysteine 10^{-2}M	lysine 10^{-2}M	histidine 10^{-2}M	leucine 10^{-2}M
**A population**													
**Speed**													
SF	-	ns	ns	ns	-	↗	ns	↗	↗	↗	ns	ns	ns
F2	-	-	↘	-	-	ns	ns	-	-	ns	↗	ns	ns
CF	ns	ns	↘	ns	ns	ns	ns	ns	ns	ns	ns	↘	ns
**Position**													
SF	-	ns	ns	ns	-	ns	ns	ns	↘	ns	ns	ns	ns
F2	-	-	↗	-	-	↗	↗	-	-	ns	ns	↗	ns
CF	↗	↗	↗	↗	ns	↗	↗	↗	↗	↗	ns	↗	↗
**NbrtX**													
SF	-	ns	ns	ns	-	ns	↗	↗	↗	ns	ns	↗	↗
F2	-	-	↘	-	-	ns	ns	-	-	ns	ns	ns	ns
CF	ns	↘	↘	↘	ns	↘	↘	ns	↘	ns	↘	↘	↘
**NbrtY**													
SF	-	↗	ns	ns	-	ns	↗	↗	↗	↗	ns	↗	ns
F2	-	-	↘	-	-	ns	↗	-	-	ns	↗	ns	ns
CF	ns	↘	↘	↘	ns	ns	ns	ns	ns	ns	↘	↘	ns
**Patterns**													
SF	-	ns	ns	ns	-	ns	change	change	ns	change	ns	ns	ns
F2	-	-	change	-	-	ns	change	-	-	change	ns	change	ns
CF	change	change	change	change	ns	ns	change	ns	change	change	change	change	ns
**B individual**													
**Score > 1.5**													
SF	-	3.2%	19.3%	42%	-	25%	41.1%	18.7%	37.5%	51%	21%	42.1%	21%
F2	-	-	50%	-	-	31.3%	18.8%	-	-	55%	18.7%	55%	36.9%
CF	25%	81.3%	73.2%	56.3%	18.7%	6.2%	35.7%	31%	43.8%	39.5%	48.4%	69.2%	28%
